# Predictive factors for the success of McRoberts’ manoeuvre and suprapubic pressure in relieving shoulder dystocia: a cross-sectional study

**DOI:** 10.1186/s12884-016-1125-3

**Published:** 2016-10-29

**Authors:** Zara Lin Zau Lok, Yvonne Kwun Yue Cheng, Tak Yeung Leung

**Affiliations:** Department of Obstetrics and Gynaecology, The Chinese University of Hong Kong, Prince of Wales Hospital, 30-32 Ngan Shing Street, Shatin, Hong Kong SAR, China

**Keywords:** McRoberts’ manoeuvre, Suprapubic pressure, Shoulder dystocia, Instrumental delivery, Vacuum extraction

## Abstract

**Background:**

McRoberts’ and suprapubic pressure are often recommended as the initial choices of manoeuvres to manage shoulder dystocia, as they are believed to be less invasive compared to other manoeuvres. However, their success rates range from 23 to 40 %. This study aims to investigate the predictive factors for the success of McRoberts’ manoeuvre with or without suprapubic pressure (M+/−S).

**Methods:**

All cases of shoulder dystocia in a tertiary hospital in South East Asia were recruited from 1995 to 2009. Subjects were analysed according to either ‘success’ or ‘failure’ of M+/−S. Maternal and fetal antenatal and intrapartum factors were compared by univariate and multivariate analysis.

**Results:**

Among 198 cases of shoulder dystocia, M+/−S as the primary manoeuvre was successful in 25.8 %. The other 74.2 % needed either rotational or posterior arm manoeuvres or combination of manoeuvres. Instrumental delivery was the single most significant factor associated with an increased risk of failed M+/−S on logistic regression (*p* < 0.001, OR 4.88, 95 % CI 2.05–11.60). The success rate of M+/−S was only 15.0 % if shoulder dystocia occurred after instrumental delivery but was 47.7 % after spontaneous vaginal delivery.

**Conclusions:**

When shoulder dystocia occurs after instrumental vaginal delivery, the chance of failure of M+/−S is 85 %, which is 4.7 times higher than that after spontaneous vaginal delivery. Hence all operators performing instrumental delivery should be proficient in performing all manoeuvres to relieve shoulder dystocia when M+/−S cannot do so.

## Background

Shoulder dystocia is an uncommon obstetric emergency with a quoted incidence ranging from 0.58 to 0.7 % [1]. In a brief few minutes it can lead to fetal morbidity and mortality [2–4], and in attempt to expedite delivery also maternal morbidity such as postpartum haemorrhage and major perineal tears [5]. An attempt to identify predictors of shoulder dystocia to sanction the option of caesarean section has only returned risk factors with low predictive value [6, 7]. Due to the unpredictable and difficult nature of shoulder dystocia many professional bodies advocate regular simulated training with high fidelity simulators and use of algorithms [1, 8–10] and mnemonics such as ‘HELPERR’ and ‘BE CALM’ (Table 1) [11, 12] to improve the outcome of said condition when it does occur. Common elements between the various algorithms are to perform McRoberts’ manoeuvre and suprapubic pressure in the first instance, and a failure of these two methods should be followed by other manoeuvres such as rotational methods, posterior arm delivery and all- fours manoeuvre. More injurious manoeuvres such as clavicular fracture, symphysiotomy and Zavanelli manoeuvres are rarely used due to the high success rates of the less invasive manoeuvres [5, 13, 14].

**Table 1:** The details of HELPERR and BE CALM Mnemonics

HELPERR Mnemonics	BE CALM Mnemonics
Help: call for help	Breathe, do not push
Evaluate for episiotomy	Elevate the legs into McRoberts position
Legs: McRoberts position	Call for help
Pressure: Suprapubic pressure	Apply suprapubic pressure
Enter manoeuvres: perform internal rotation	EnLarge the vaginal opening: perform episiotomy if more room is needed for manoeuvres
Remove the posterior arm	Manoeuvres deliver the posterior arm or perform rotational manoeuvres
Roll the patient onto all fours	

McRoberts’ and suprapubic pressure are often recommended as the initial choices of manoeuvres because they are believed to be less invasive compared to other manoeuvres, which require the insertion of operators’ hands into the vagina. However, even well conducted McRoberts’ manoeuvre and suprapubic pressure do not guarantee success of delivery without injury. Studies from previous cohorts have claimed success rates ranging from 23.2 to 58 % for McRoberts’ manoeuvre alone or in combination with suprapubic pressure [5, 13, 15]. In order to perform McRoberts’ manoeuvre and suprapubic pressure correctly and effectively, there must be adequate staff available, including two persons to hyper-flex the maternal hips (one on each side), a third one to apply suprapubic pressure, and another one to apply traction on the fetal head. Failure to summon additional staff to assist immediately may delay the use of more effective rotational methods or posterior arm delivery, and increase the risk of fetal hypoxia [2]. Thus it is necessary to review the usefulness of these frequently used first-line manoeuvres, by investigating which factors contributing to their success or failure, and in doing so decide how strongly one must adhere to a fixed protocol in each scenario.

The root of this current review stems from two previous studies published by our group regarding the head-to-shoulder delivery interval and perinatal outcomes of shoulder dystocia [2, 3]. We subsequently noted that the 25 % success rate of McRoberts’ manoeuvre and suprapubic pressure in our Asian centre was similar to a recently published paper from the Netherlands (23.8 %) [16], but was significantly lower than that reported in other predominantly Caucasian centres such as that of MacKenzie et al. and Gherman et al. (46 and 42 % respectively) [13, 15]. There are only a few reports on the success rate of McRoberts’ manoeuvre and suprapubic pressure in relieving shoulder dystocia. This led us to investigate the predictive factors for the success of McRoberts’ manoeuvre with or without suprapubic pressure (M+/−S).

## Methods

This cross-sectional study was conducted in a tertiary university hospital in Hong Kong with an annual delivery of more than 6000. All consecutive cases of shoulder dystocia reported from 1995 to 2009 inclusively were identified from our hospital electronic database. As per our previous studies [2, 3], shoulder dystocia was defined as either a need to perform an additional obstetric manoeuvre in addition to downward traction of the fetal neck or when the head to body delivery interval was longer than 1 min [17]. Cases were only included for analysis if McRoberts’ manoeuvre with or without suprapubic pressure (M+/−S) was the first manoeuvre performed, and documentation was available regarding the management of dystocia. Cases of intrauterine fetal death or fetal malformations were excluded. Our unit protocol for the management of shoulder dystocia was based on and similar to the Green Top Guideline on shoulder dystocia published by the Royal the College of Obstetricians & Gynaecologists [1]. All midwives and obstetricians took part in annual drills on the management of shoulder dystocia. In all cases, unless otherwise stated, McRoberts’ combined with suprapubic pressure was the first manoeuvre attempted. If this failed other manoeuvres would be attempted based on the operators’ experience at the time. A nurse was always designated to document time sequences, in particular head and body delivery times. At least one obstetrician and one paediatrician would attend the cases at the time of diagnosis. All instrumental deliveries in our unit were conducted by obstetricians. All cases of shoulder dystocia were audited in a monthly meeting and logged. Unless otherwise stated, M+/−S in this study refers to the use of McRoberts’ manoeuvre with or without suprapubic pressure, as our unit protocol requires the two to be carried out simultaneously.

Identified cases were traced and medical records were searched for factors of interest, which included both maternal and neonatal antenatal and intrapartum characteristics. Maternal age was noted and advanced maternal age was defined as 35 years or above. Maternal height was defined as short stature if 150 cm or less. Maternal body weight at booking and at the time of delivery was recorded to calculate the body mass index (BMI) at the two corresponding time points, and women were classified as obese according to the World Health Organization cut-off of 30 kg/m^2^ [18]. Maternal ethnicity, parity, history of shoulder dystocia and presence of diabetes mellitus were also noted. Intrapartum characteristics included the onset of labour, mode of delivery, use of epidural analgesia, and duration of the second stage of labour. Prolonged second stage was defined as more than 60 min. Neonatal characteristics of note included gestational age, neonatal sex and birth weight.

Analysis was done using IBM® SPSS® Statistics version 22 (IBM, N.Y., USA). Maternal antenatal and intrapartum characteristics and neonatal demographics were analysed in relation to the success of M+/−S using Chi-square test for categorical independent variables and independent *t*-test for continuous independent variables. All variables with *p* <0.2 on univariate analysis were further recruited for multivariate analysis. The level of statistical significance was set at *p* < 0.05.

Ethical approval for this study was obtained from the Institutional Review Board ‘Joint The Chinese University of Hong Kong – New Territories East Cluster Clinical Research Ethics Committee’ (Ref. No. CRE-2010.029) on 04 February 2010.

## Results

A total of 210 cases of shoulder dystocia were identified amongst the 62,295 singleton vaginal deliveries from 1995 to 2009 inclusively. The incidence of 0.34 % is comparable to those reported worldwide [19]. Twelve cases were excluded from analysis, as M+/−S was not the first manoeuvre performed. Of the remaining 198 cases, majority of the women in this cohort were Chinese (94.9 %), with the remaining 5.1 % being of South Asian ethnicity including Filipino, Indian, Pakistani, Indonesian, Thai and Vietnamese. The mean maternal age was 31.0 ± 4.9 years and 77.2 % were aged less than 35 years at the time of delivery. There were 96 nulliparous and 102 multiparous women, and none of them had previous history of shoulder dystocia. One hundred thirty-three (67.2 %) needed instrumental delivery (130 vacuum extractions and three forceps deliveries), and all were performed by obstetricians. The indication for instrumental delivery was either ‘fetal distress’ (56 cases, 42.1 %) or ‘prolonged second stage of labour’ (77 cases, 57.9 %). Fifty-one cases (25.8 %) were delivered successfully with M+/−S; of this 21 (41.2 %) were by midwives alone. For the other 147 cases who had failed M+/−S, additional obstetric manoeuvres such as rotational or posterior arm delivery or combination of the two were performed to achieve delivery as reported in our previous study [3]. No case needed all-fours manoeuvre, symphysiotomy, Zavanelli manoeuvre or caesarean section, and all babies were delivered live at birth. There were 4 cases of brachial plexus injury (7.8 %) (*N* = 4) in the success group and 10 (6.8 %) in the failed group (*p* = 0.946). Other perinatal outcomes were reported separately in our previous studies [2, 3].

Table 2 listed the comparison of maternal factors, intrapartum factors and neonatal factors between the success group and the failed M+/−S group. There were no statistical differences between the failed group and the successful group in terms of Chinese ethnicity (95.2 % vs. 94.1 %), maternal age (31 year old vs. 30 year old), maternal height (156 cm vs. 156 cm), BMI at booking (24.1 kg/m^2^ vs. 24.8 kg/m^2^), proportion of nulliparity (51.7 % vs 39.2 %), and presence of diabetes mellitus (8.8 % vs. 11.8 %). However, women in the failed group were statistically significantly lighter than those in the success group in terms of their mean maternal body weight at delivery (68.1 kg vs. 71.7 kg, *p* = 0.034), and their mean BMI at delivery (27.8 kg/m^2^ vs. 29.2 kg/m^2^, *p* = 0.015).. The proportion of obese women (BMI at delivery of 30 kg/m^2^ or more) was significantly lower in the failed group (21.1 % vs. 33.3 %; *p* = 0.049).

**Table 2:** Maternal, Fetal Antenatal and Intrapartum Characteristics of Women with Shoulder Dystocia (*N* = 198)

McRoberts’ manoeuvre with and without suprapubic pressure				
		Fail (*n* = 147)	Success (*n* = 51)	*p*
Maternal Characteristics				
Ethnicity	Chinese	140 (95.2)	48 (94.1)	
	Other Asian	7 (4.8)	3 (5.9)	0.753
Maternal Age	Mean ± SD	31 ± 4.8	30 ± 5.2	0.300
	<35 years	113 (76.9)	40 (78.4)	
	≥35 years	34 (23.1)	11 (21.6)	0.819
Maternal Height (cm)^a^	Mean ± SD	156 ± 6.0	156 ± 5.5	0.737
	>150	120 (81.6)	45 (88.2)	
	≤150	24 (16.3)	4 (7.8)	0.144
Maternal Weight at delivery (kg)		68.1 ± 9.9	71.7 ± 9.2	*0.034*
Maternal BMI at booking (kg/m^2^)^b^	Mean ± SD	24.1 ± 3.9	24.8 ± 3.5	0.312
	<30	128 (87.1)	45 (88.2)	
	≥30	14 (9.5)	2 (3.9)	0.232
Maternal BMI at delivery (kg/m^2^)^c^	Mean ± SD	27.8 ± 3.4	29.2 ± 3.5	*0.015*
	<30	108 (72.5)	29 (56.9)	
	≥30	31 (21.1)	17 (33.3)	*0.049*
Parity	Nulliparous	76 (51.7)	20 (39.2)	
	Multiparous	71 (48.3)	31 (60.8)	0.124
Pre-existing/Gestational Diabetes Mellitus	No	134 (91.1)	45 (88.2)	
	Yes	13 (8.8)	6 (11.8)	0.542
Intrapartum Characteristics				
Onset of Labour	Spontaneous	112 (76.2)	43 (84.3)	
	Induced	35 (23.8)	8 (15.7)	0.285
Duration of 2^nd^ stage (minutes)^d^	Mean ± SD	55 ± 41.2	42 ± 40.7	0.056
	Normal (≤60 min)	84 (57.1)	37 (72.5)	
	Prolonged (>60 min)	61 (41.5)	13 (25.5)	*0.043*
Mode of Delivery	Spontaneous vaginal	34 (23.1)	31 (60.8)	
	Instrumental	113 (76.9)	20 (39.2)	*<0.001*
Epidural	With	25 (17.0)	6 (11.8)	
	Without	122 (83.0)	45 (88.2)	0.375
Infant Characteristics				
Gestation (weeks)		39 ± 1.2	39 ± 1.2	0.658
Sex	Male	91 (61.9)	28 (54.9)	
	Female	56 (38.1)	23 (45.1)	0.379
Body Weight (kg)	Mean ± SD	3.78 ± 0.39	3.87 ± 0.38	0.178
	<4 kg	102 (69.4)	33 (64.7)	
	≥4 kg	45 (30.6)	18 (35.3)	0.536

Note: Data presented as n (%) or mean ± standard deviation; *SD* standard deviation

a: 5 missing data; b:9 missing data; c:13 missing data; d:3 missing data

For intrapartum factors of those who failed M+/−S, 34 (23.1 %) followed spontaneous delivery of the head, while 113 (76.9 %) followed instrumental delivery, (111 vacuum extractions and two forceps deliveries). Instrumental delivery was significantly higher in the failed group (76.9 %) when compared to the success group (39.2 %; *p* < 0.001). The second stage of labour was more likely to be prolonged in the failed group (41.5 % vs. 25.5 %; *p* = 0.043), but there was no difference in term of induction of labour (23.8 % vs. 15.7 %; *p* = 0.285) or the use of epidural analgesia (17.0 % vs. 11.8 %; *p* = 0.375). There was also no difference in terms of gestational age (39 weeks vs. 39 weeks, *p* = 0.658), infant sex (p = 0.379) or body weight at birth (3.78 kg vs. 3.87 kg, *p* = 0.178).

After multivariate analysis of variables associated with failure of M+/−S, only instrumental delivery remained to be the single significant factor for the failure of M+/−S in shoulder dystocia (*p* <0.001, OR 4.88, 95 % CI 2.05–11.60; Table 3). The success rate of M+/−S in the event of shoulder dystocia was only 15.0 % if occurring after instrumental delivery, but was 47.7 % if occurring after natural delivery of the fetal head.

**Table 3:** Multivariate logistic regression of factors associated with failure of McRoberts’ manoeuvre and suprapubic pressure

	*p*	OR (95 % CI)
Parity	0.641	0.80 (0.32–2.03)
Short maternal height (≤150 cm)	0.146	0.31 (0.07–1.50)
Maternal weight at delivery (kg)	0.619	1.03 (0.93–1.13)
Maternal BMI at delivery (kg/m^2^)	0.279	0.85 (0.63–1.14)
Obesity (BMI ≥30 kg/m^2^) at delivery	0.663	0.75 (0.20–2.76)
Instrumental delivery	*<0.001*	*4.88 (2.05–11.60)*
Duration of second stage	0.619	1.00 (0.98–1.11)
Prolonged second stage (>60mins)	0.612	0.64 (0.12–3.55)
Birth weight of baby	0.828	1.00 (1.00–1.001)

## Discussion

Although many factors such as maternal weight, height, BMI [20], and infant birth weight [7] may affect the success of McRoberts’ manoeuvre and suprapubic pressure (M+/−S), we have identified instrumental delivery to be the single most significant factor which increases the risk of failure of M+/−S during shoulder dystocia after multi-variate analysis (*p* < 0.001, OR 4.88). We did not find statistical relationship between the success of M+/−S and parity, maternal body weight, height or BMI, and infant birth weight. Although the presence of prolonged second stage of labour and maternal BMI ≥30 kg/m^2^ at the time of delivery was shown significant in univariate analysis, they lost significance on multivariate analysis.

The 25.8 % success rate of M+/S in our Asian centre was similar to a midwifery cohort from the Netherlands reported by Kallianidis et al. (23.8 %) [16], but was significantly lower than that of MacKenzie et al. and Gherman et al. (46 and 42 % respectively) [13, 15]. Notable differences between our cohort and those of Gherman et al. [15] and MacKenzie et al. [13] are that, Gherman et al’s consisted mainly of multiparous women (86 %) with spontaneous vaginal deliveries (90.4 %) and ethnically Caucasian, as opposed to our cohort of 52 % multiparous and 48 % nulliparous women, who delivered predominantly by instrumental delivery (67.2 %), and were ethnically Asian. As instrumental delivery appears to be a key factor in the failure of M+/−S, that Gherman et al. had fewer instrumental deliveries may account for the difference in the success rate. Likewise, only 35.4 % had instrumental delivery in MacKenzie et al’s study [13]. On the other hand, in the Dutch cohort reported by Kallianidis et al., 92 % were multiparous after spontaneous birth but they yielded only a success rate of 23.8 % [16].

Although instrumental delivery is a known risk factor causing shoulder dystocia [21, 22], its association with failure of M+/−S is a new finding and the underlying mechanism is not well known. In spontaneous vaginal delivery, uterine contractions and maternal effort cause gradual and synchronized descent of the fetal head and shoulders. Contrasting instrumental delivery where the head is pulled out by a stronger force in a relatively short period of time resulting in rapid descent of the fetal head and shoulders through the pelvis [20, 23]. It was estimated that during a vacuum extraction, the traction force on the fetus is up to 294 N [24], nine times higher than the usual force of spontaneous delivery [25]. This may result in the shoulders, especially the posterior shoulder, not descending along the curved sacral path synchronously with the pulled head. After delivery of the head, the posterior shoulder may still be well above the sacral promontory or mid-level of sacrum, hence is unresponsive to McRoberts’ manoeuvre, which improves the relative shoulder-to-pelvic dimension by only 1 cm [26]. The mal-descended shoulders may also fail to respond to suprapubic pressure, which only attempts to rotate the anterior, and not the posterior shoulder. Therefore, instrumental delivery increases the risk of shoulder dystocia occurrence in addition to rendering McRoberts’ manoeuvre and suprapubic pressure unsuccessful in such circumstances.

**Fig. 1:**
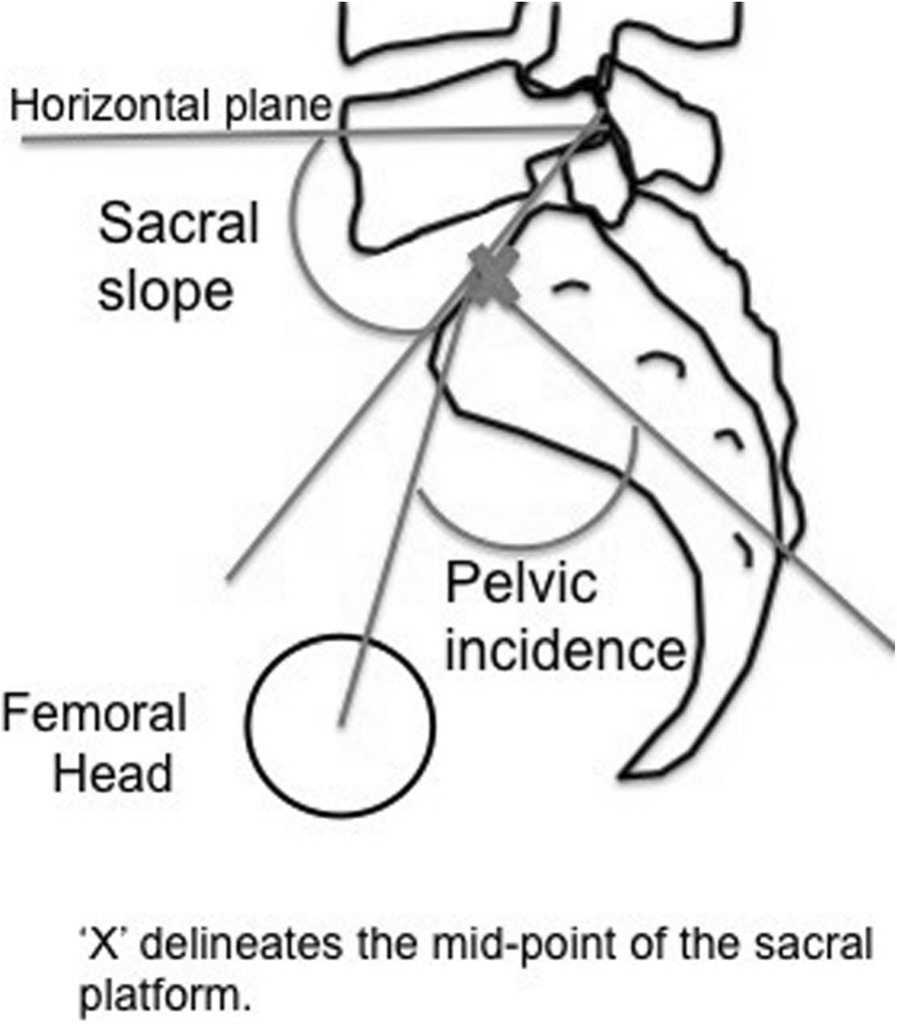
Measurement of sacral slope and pelvic incidence angles

**Fig. 2:**
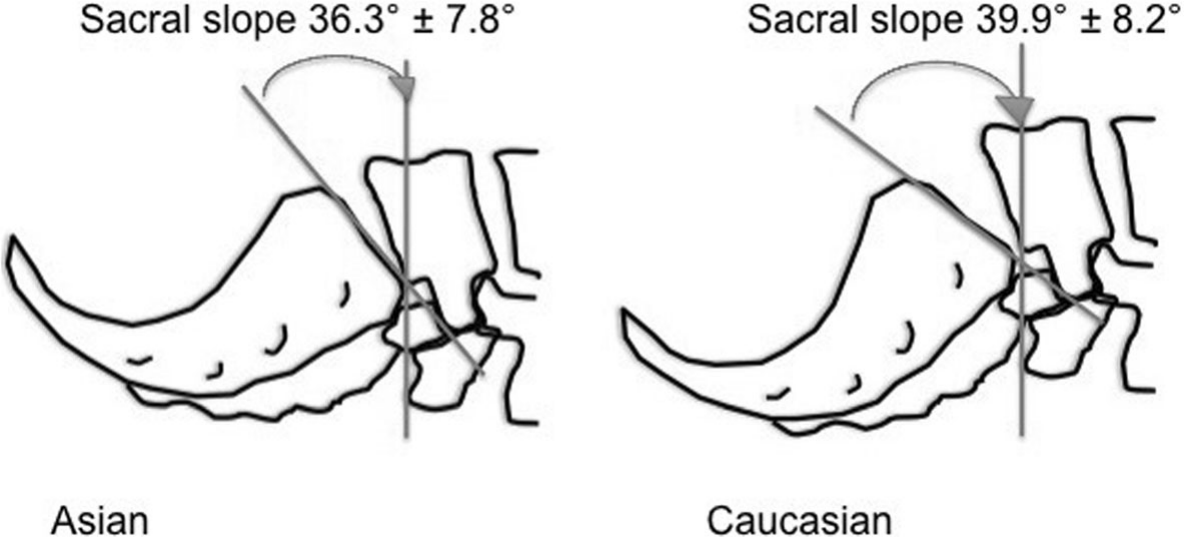
Difference in sacral slope angles between Asian and Caucasian pelvises. - Larger sacral slope angle in theory allows greater degree of movement of Caucasian pelvises. Based on data from Zárate-Kalfópulos B, Romero-Vargas S, Otero-Cámara E, Correa VC, Reyes-Sánchez A. Differences in pelvic parameters among Mexican, Caucasian, and Asian populations. J Neurosurg Spine. 2012;16:516–519 [23]

**Fig. 3:**
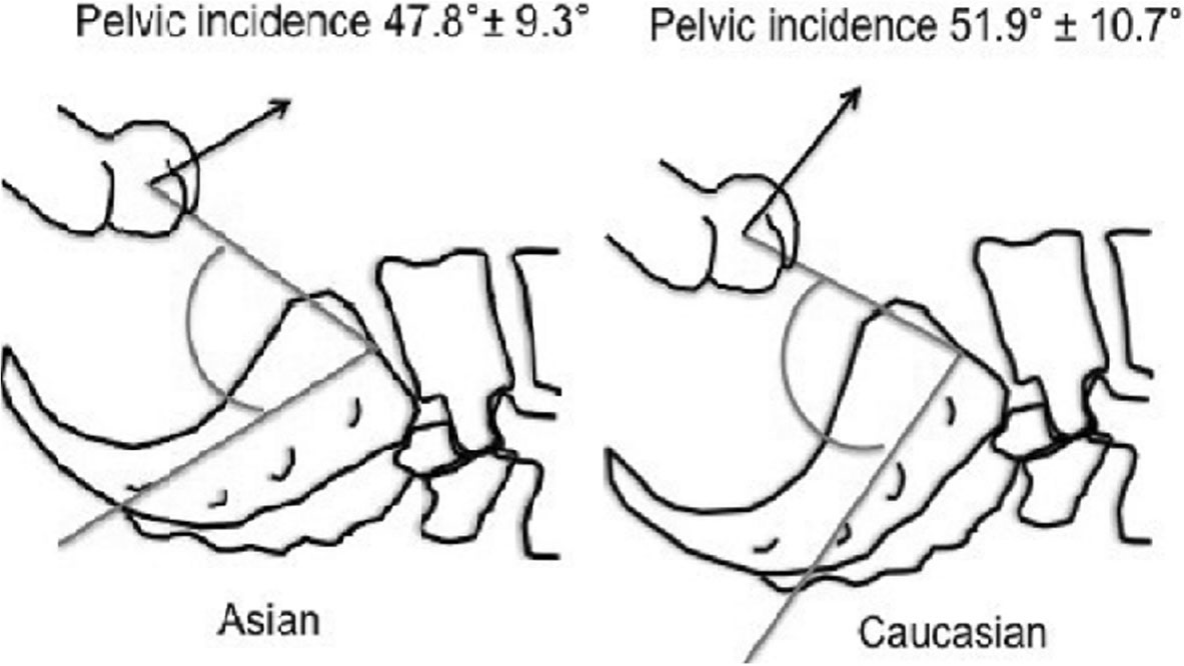
Difference in pelvic incidence angles between Asian and Caucasian pelvises and direction of force (as indicated by *black arrows*) needed to achieve pelvic rotation. - Based on data from Zárate-Kalfópulos B, Romero-Vargas S, Otero-Cámara E, Correa VC, Reyes-Sánchez A. Differences in pelvic parameters among Mexican, Caucasian, and Asian populations. J Neurosurg Spine. 2012;16:516–519 [23]

Ethnic differences in pelvic anatomy may affect the success rate of M+/−S, although even among different Caucasian cohorts the reported success rate varies from 23.8 % [16] to 46 % [13]. In an analysis comparing radiological pelvic parameters between Mexicans, Caucasians and Asians it was found that Asians have a lesser sacral slope and pelvic incidence [23]. The sacral slope is the angle created by the horizontal plane and sacral platform when standing, or by the vertical plane when supine. It is a dynamic parameter and changes with posture. The pelvic incidence is the angle created by a line perpendicular to the sacral platform at its midpoint and the line that connects this midpoint with the midpoint of the femoral head (Fig. 1). It is a morphological parameter and remains constant for any given person. A lesser pelvic incidence corresponds to less lumbar lordosis, and with a lesser sacral slope implies a more limited range of anterior and posterior pelvic tilt [26] (Fig. 2). In the study by Zárate-Kalfópulos et al. the reported sacral slope for Asians was 36.3 and 39.9° for Caucasians. The pelvic incidence for Asians was 47.8 and 51.9° for Caucasians. The ethnic difference in pelvic incidences may also point to a necessary difference in direction of thigh and femur elevation in order to achieve maximum pelvic rotation (Fig. 3). Thus when McRoberts’ manoeuvre is performed in Asians, there may be less flexibility for rotation and the mechanism of movement may be different. Hence the dramatic change in angles as suggested by Gherman et al. [15] and Gonik et al. [27] in the pelvimetry models may not be achieved, leading to a failure of McRoberts’ manoeuvre.

The practice of the accouchers may also affect the success of McRoberts’ and suprapubic pressure. In Hong Kong, midwives conduct all spontaneous vaginal deliveries. When they encounter shoulder dystocia, they will only perform McRoberts’ and suprapubic pressure whilst waiting for the obstetricians to arrive. Hence midwives may use more time and effort in performing McRoberts’ manoeuvre, contributing to its success. In contrast, all instrumental deliveries are conducted by obstetricians, who may adopt other manoeuvres earlier, either due to their familiarity and knowledge of the other manoeuvres, or to their reluctance for forceful traction of fetal neck during McRoberts’.

Gonik et al’s mathematical model suggests that McRoberts’ manoeuvre may reduce the traction force needed to achieve delivery by 50 % [27]. However, it does not reduce the risk of brachial plexus injury in clinical practice, as shown by MacKenzie et al’s study where the increased use of McRoberts’ actually resulted in an increase in brachial plexus injury [13]. The injury may have resulted from undetected failure of McRoberts’ manoeuvre in cases where there has been delayed descent of the fetal shoulders, but in an effort to achieve delivery accouchers might have applied an excessive traction during a persistent McRoberts’ procedure, resulting in injury [28]. Hence, proper training is essential to ensure safe relief of shoulder dystocia [29], and accouchers should have regular high fidelity training to achieve competence in conducting all manoeuvres [30]. McRoberts’ manoeuvre should still be performed firstly, as it is simple to learn and perform, and if it succeeds can eliminate the need for internal manoeuvres, which may increase the risk of fetal injury under inexperienced hands [3].

Our finding of the association between instrumental delivery and failure of McRoberts’ manoeuvre and suprapubic pressure may be controversial in clinical practice. As the failure rate after instrumental delivery is high, it is debatable whether the operator should recourse immediately to rotational methods, which is associated with a higher success rate and low morbidity [3]. By shortening the head-to-shoulder delivery interval, the chance of hypoxic ischemic injury may be reduced [2]. However, if the mechanism of the failure is the delayed decent of the posterior shoulder, it is debatable whether more time should be allowed for the shoulder to descend spontaneously before starting aggressive intervention.

Our study is limited by an ethnically Asian cohort without Caucasians for direct comparison. Whether our findings are applicable to other ethnic groups need further investigation, as their pelvic configuration may be different [31]. However, our study is strengthened by a sizable cohort, comprehensive recruitment and uniform management of shoulder dystocia through the years due to routine drills and monthly audit reviews. We did not further separate the success rates for McRoberts’ manoeuvre from suprapubic pressure as both were commonly performed simultaneously, making the estimation of individual’s success rate imprecise.

## Conclusion

When shoulder dystocia occurs after instrumental vaginal delivery, the chance of failure of McRoberts’ manoeuvre is 85 %, which is a significant 4.88 times higher as compared to spontaneous vaginal delivery. There should be a quick resort to other manoeuvres to expedite delivery and all operators performing instrumental delivery should be proficient in all manoeuvres. Further studies are required to substantiate the generalisability of our findings, and assess the degree of impact instrumental delivery and ethnicity has on the management of shoulder dystocia.

## Abbreviations

BMI: Body mass index
CI: Confidence interval
M+/−S: McRoberts’ manoeuvre with or without suprapubic pressure
OR: Odds ratio
vs.: Versus
